# Supplementary material for: Melatonin in Glaucoma: Integrative Mechanisms of Intraocular Pressure Control and Neuroprotection
Source: Biomedicines. 2025 May 16;13(5):1213. doi: 10.3390/biomedicines13051213 (PMC12108883; doi:10.3390/biomedicines13051213)
Supplement: Supplementary file 1 [file biomedicines-13-01213-s001.zip › Supplementary_Table_S1_Melatonin_IOP_NoYear.pdf]

Supplementary Table S1: Summary of Evidence Linking Melatonin to Intraocular Pressure Reduction

Table S1: Summary of Studies Demonstrating the IOP-Lowering Effects of Melatonin

author	Type of Study	Study Sample	Methods and intervention	Administration method	comparison	outcome	conclusion
Samples, Krause, and Lewy Curr Eye Res. 1988;7:649-653	Experimental study (human)	Healthy human volunteers	Oral melatonin administration; exposure to bright/dim light to assess melatonin suppression and IOP effects.	Oral melatonin administration	Bright light exposure vs. melatonin administration	Melatonin lowered IOP; suppression of melatonin via bright light attenuated IOP decrease.	Melatonin may play a role in IOP reduction and could be a potential therapeutic agent for glaucoma.
Viggiano, S R et al. Ophthalmology. 1994;101:326-331	Randomized, masked crossover study	19 healthy human volunteers	Oral melatonin administered during the day; aqueous humor flow measured by fluorophotometry for 8 hours	Oral melatonin administration	Compared melatonin treatment with placebo	No significant difference in aqueous humor flow between melatonin and placebo ($P = 0.4$)	Daytime melatonin levels comparable to nocturnal peaks do not suppress aqueous humor formation, suggesting melatonin is not the primary regulator of circadian aqueous humor flow.
Pintor, J et al. Eur J Pharmacol. 2001;416:251-254	Experimental Study	New Zealand white rabbits	Tested melatonin and the selective MT3 receptor agonist 5-MCA-NAT for IOP reduction; used luzindole to confirm receptor involvement.	Topical application of melatonin and 5-MCA-NAT	Comparison of melatonin and 5-MCA-NAT; effect blocked by luzindole.	5-MCA-NAT reduced IOP by 43%, melatonin by 24%, both in a receptor-dependent manner.	Melatonin and 5-MCA-NAT lower IOP via MT3 receptors, supporting a role for melatonin in circadian IOP regulation.
Janet B. Serle et al. J Glaucoma. 2004;13:385-388	Experimental Study	8 glaucomatous cynomolgus monkeys	Topical application of 5-MCA-NAT (2%) twice daily for 5 days; IOP measured at multiple time points.	Topical application (5-MCA-NAT, 2%) twice daily for 5 days	Comparison with vehicle-treated eyes	5-MCA-NAT significantly reduced IOP (10% on day 1, 15% on day 3, 19% on day 5); effects lasted $\geq$ 18 hours.	5-MCA-NAT, a melatonin MT3 receptor agonist, effectively lowers IOP in glaucomatous monkey eyes and may have clinical potential for glaucoma treatment.
Alarma-Estrany, Pilar et al. J Pineal Res. 2008;45:468-475	Experimental Study (Animal)	New Zealand white rabbits	Evaluated the effect of the MT2 melatonin receptor agonist IIK7 on IOP and the involvement of the sympathetic nervous system. Used receptor antagonists and sympathectomy to analyze mechanisms.	Topical IIK7 application	Compared effects of IIK7, melatonin, and β-adrenergic agonists (terbutaline, salbutamol).	IIK7 reduced IOP by 38.5%, melatonin by 20.2%. Effects were blocked by MT2 antagonists and reduced after chemical sympathectomy. β-adrenergic agonists enhanced IIK7's effect.	MT2 melatonin receptors contribute to IOP reduction, likely by decreasing aqueous humor production. The sympathetic nervous system modulates this effect, enhancing its potential for glaucoma treatment.

Continued on next page

Table S1 Continued

**author**	**Type of Study**	**Study Sample**	**Methods and intervention**	**Administration method**	**comparison**	**outcome**	**conclusion**
Ismail, Salah A, and Hany A Mowafi. Anesth Analg. 2009;108:1146-1151	Randomized controlled trial (human)	40 patients undergoing cataract surgery	Evaluated effects of oral melatonin (10 mg) 90 min before surgery on IOP, pain, anxiety, and surgical conditions	Oral melatonin (10 mg) before cataract surgery	Compared melatonin group vs. placebo group	Melatonin significantly reduced IOP (from 17.9 to 13.8 mmHg), anxiety, and pain, and improved surgical conditions	Melatonin premedication enhances analgesia, reduces anxiety, and lowers IOP, making it beneficial for cataract surgery under topical anesthesia
Alarma-Estrany, Pilar et al. J Pharmacol Exp Ther. 2011;337:703-709	Experimental Study	160 ocular normotensive rabbits	Synthesis and testing of novel melatonin analogs (INS48848, INS48862, INS48852) for IOP reduction; administration at doses of 0.1 mM and 1 mM	Topical application of novel melatonin analogs (INS48848, INS48862, INS48852)	Compared with previous melatonin receptor agonists (5-MCA-NAT, IIK7); receptor antagonist studies for mechanism identification	All tested melatonin analogs reduced IOP; INS48848 (MT3) lowered IOP by 36%, INS48862 (MT2) by 24%, and INS48852 (MT2) by 30%; dose-dependent effects	Novel melatonin analogs effectively reduce IOP, with INS48848 acting via MT3 receptors and INS48862/INS48852 via MT2 receptors. These compounds may serve as leads for glaucoma treatment.
Crooke, Almudena et al. J Pineal Res. 2012;52:265-270	Experimental Study (Animal & Cellular)	New Zealand white rabbits; cultured rabbit nonpigmented ciliary epithelial (NPE) cells	Investigated the effect of 5-MCA-NAT on carbonic anhydrases (CAs) CAII and CAXII in IOP regulation; used qPCR, immunohistochemistry, and IOP monitoring	Topical 5-MCA-NAT applied to New Zealand white rabbits	Compared 5-MCA-NAT treatment with control and Trusopt® (a CA inhibitor)	5-MCA-NAT reduced IOP by 51.3% (maximal at 3h), with sustained effects for up to 96h. It downregulated CAII and CAXII at mRNA and protein levels in NPE cells.	5-MCA-NAT lowers IOP by downregulating CAII and CAXII, suggesting its potential as a long-term therapy for glaucoma.
Crooke, Almudena et al. J Pharmacol Exp Ther. 2013;346:138-145	Experimental Study	Cultured rabbit nonpigmented ciliary epithelial cells; normotensive rabbits	Investigated effects of melatonin and 5-MCA-NAT on $\beta 2/\alpha 2$A-adrenergic receptor mRNA and protein expression; tested ocular hypotensive effects in rabbits pretreated with timolol or brimonidine.	Topical application of melatonin and 5-MCA-NAT	Compared effects of melatonin and 5-MCA-NAT with timolol and brimonidine alone.	5-MCA-NAT and melatonin modulated adrenergic receptor expression; pretreatment enhanced IOP reduction with timolol (by 14-16.75%) and brimonidine (by 29.26-39.07%).	Melatonin and 5-MCA-NAT enhance the ocular hypotensive effects of adrenergic drugs, suggesting a synergistic role in glaucoma treatment.
Dortch-Carnes, Juanita, and Gianluca Tosini. Exp Eye Res. 2013;107:1-10	Experimental Study	Isolated human non-pigmented ciliary epithelial (hNPCE) cells	Evaluated effects of melatonin and receptor agonists (5-MCA-NAT, IIK7) on SNP-released nitric oxide (NO) and cGMP production in aqueous humor-producing cells	Melatonin, 5-MCA-NAT, IIK7 on isolated human NPCE cells	Compared melatonin, 5-MCA-NAT, and IIK7; tested effects with MT1/MT2 antagonists (luzindole, 4P-PDOT)	Melatonin, 5-MCA-NAT, and IIK7 reduced NO and cGMP levels in a concentration-dependent manner; effects were blocked by MT2 antagonists	MT2 receptors mediate melatonin-induced reduction of NO and cGMP, suggesting a role in regulating aqueous humor production and IOP

Continued on next page

Table S1 Continued

author	Type of Study	Study Sample	Methods and intervention	Administration method	comparison	outcome	conclusion
Pescosolido, Nicola et al. Ophthalmic Physiol Opt. 2015;35(2):201–205	Pilot Prospective Study (Human)	10 glaucoma patients (POAG)	Evaluated the effect of oral agomelatine (25 mg/day) for 30 days on IOP in patients on maximum tolerated medical therapy	Oral agomelatine (25 mg/day for 30 days)	IOP at baseline vs. after 15 and 30 days of agomelatine treatment	Agomelatine reduced IOP by ˜30% at both 15 and 30 days, with a stable effect.	Oral agomelatine significantly reduces IOP in glaucoma patients unresponsive to conventional therapy, suggesting a potential new treatment approach.
Martínez-Águila, Alejandro et al. J Pharmacol Exp Ther. 2016;357:293-299	Experimental Study	C57BL/6J control mice and glaucomatous DBA/2J mice	Evaluated the effects of melatonin and 5-MCA-NAT on IOP using TonoLab tonometry; tested receptor antagonists.	Topical application of melatonin and 5-MCA-NAT	Compared effects in control vs. glaucomatous mice; tested receptor antagonists.	Melatonin reduced IOP by 19.4% (control) and 32.6% (glaucoma); 5-MCA-NAT had similar effects and stopped IOP progression.	Melatonin and 5-MCA-NAT effectively lower IOP, with stronger effects in glaucoma. 5-MCA-NAT may prevent IOP progression.
Alkozi, Hanan Awad et al. Br J Pharmacol. 2020;177:2090-2105	Experimental Study (Cellular & Animal)	Cell cultures, human post-mortem eye samples, and glaucoma mouse models	Investigated adreno-melatonin receptor complexes in ion homeostasis and IOP regulation; tested melatonin and α1-adrenoceptor antagonist (prazosin) for glaucoma treatment	Topical melatonin combined with α1-adrenoceptor antagonist (prazosin)	Compared IOP in normal vs. glaucomatous eyes; tested melatonin alone vs. melatonin + prazosin	Melatonin receptors form functional complexes with α1-adrenoceptors, influencing IOP regulation; these complexes are disrupted in glaucoma. Co-administration of melatonin and prazosin significantly reduced IOP in glaucoma models.	Melatonin-adrenoceptor complexes play a key role in IOP regulation. Their disruption contributes to glaucoma, and targeting them with melatonin and prazosin offers a novel therapeutic approach.
Dal Monte, Massimo et al. Int J Mol Sci. 2020;21:9267	Experimental Study (Animal)	Hypertensive glaucoma rat model	Evaluated the hypotensive and neuroprotective effects of a topical melatonin/agomelatine formulation; assessed IOP, retinal function (ERG), gliosis-related inflammation, and apoptosis markers.	Topical instillation of a melatonin/agomelatine formulation in hypertensive glaucoma rats	Compared melatonin/agomelatine with timolol and brimonidine.	Melatonin/agomelatine reduced IOP by 60%, more effectively than timolol (32%) or brimonidine (34%). It also prevented RGC apoptosis and improved retinal function.	A topical melatonin/agomelatine formulation shows strong IOP-lowering and neuroprotective effects, suggesting its potential as a novel glaucoma treatment.

Continued on next page

Table S1 Continued

author	Type of Study	Study Sample	Methods and intervention	Administration method	comparison	outcome	conclusion
Dal Monte, Massimo et al. Diagnostics (Basel). 2020;10:138	Experimental Study (Animal)	Hypertensive glaucoma rat model	Evaluated the effect of a nanomicellar formulation of melatonin and agomelatine on IOP; assessed IOP reduction and duration of effect	Nanomicellar formulation of melatonin and agomelatine administered topically	Compared melatonin and agomelatine in saline vs. nanomicellar formulations; evaluated impact of lipoic acid addition	Nanomicellar formulation prolonged the IOP-lowering effect compared to saline. Combination of melatonin and agomelatine had enhanced and prolonged hypotensive effects, further extended by lipoic acid.	Nanomicellar formulations improve the efficacy and duration of melatoninergic compounds for IOP reduction, supporting their potential for glaucoma therapy.
Gubin, Denis et al. J Pineal Res. 2021;70:e12730	Clinical Study (Human)	115 patients with stable and advanced primary open-angle glaucoma (POAG)	Evaluated the effects of long-term (90 days) oral melatonin (2 mg, nightly) on IOP, retinal ganglion cell (RGC) function, circadian rhythms, sleep, and mood.	Oral melatonin (2 mg/night for 90 days)	Compared melatonin effects in stable vs. advanced POAG patients	Melatonin significantly lowered IOP, improved RGC function (increased PERG N95 amplitude), restored disrupted circadian rhythms, and enhanced sleep and mood, with more pronounced effects in advanced glaucoma.	Long-term melatonin administration improves IOP regulation, RGC function, and circadian stability, suggesting it as a potential therapeutic option for glaucoma management.
Giannetto, Claudia et al. J Am Vet Med Assoc. 2022;260:524-529	Experimental Study (Animal)	20 healthy adult male dogs	Evaluated the effects of 30-day oral melatonin (0.3 mg/kg daily) on tear production, IOP, and tear/serum melatonin levels.	Oral melatonin (0.3 mg/kg daily for 30 days)	Compared melatonin-treated group with placebo group.	No significant effects on tear production, IOP, or serum melatonin levels. A transient difference in tear melatonin concentration was observed only on day 1.	Long-term oral melatonin at 0.3 mg/kg/day does not significantly affect IOP, tear production, or systemic melatonin levels in healthy dogs.
Li, Ka-Lok et al. Int J Mol Sci. 2023;24:5789	Experimental Study (Porcine Model)	Porcine ciliary epithelium	Examined the effects of melatonin on aqueous humor (AH) secretion and chloride transport using Ussing chambers and fluid flow assays; evaluated receptor involvement via antagonists	Topical melatonin applied to porcine ciliary epithelium using Ussing chambers and fluid flow assays	Compared effects of melatonin application to aqueous vs. stromal side; used MT1/MT2 antagonist (luzindole) and MT3 antagonist (prazosin)	Melatonin increased Cl^- transport and AH secretion by 80%, mediated via MT3 receptors. Luzindole had no effect, while prazosin blocked the response.	Melatonin stimulates AH secretion via MT3 receptors, potentially influencing intraocular pressure regulation.

Continued on next page

Table S1 Continued

author	Type of Study	Study Sample	Methods and intervention	Administration method	comparison	outcome	conclusion
Cantarini, Mattia et al. Int J Mol Sci. 2023;24:2863	Computational & Experimental Study	Computational models of human melatonin receptors (hMT1, hMT2); normotensive and hypertensive rat models	Molecular docking and dynamics simulations to analyze melatonin and agomelatine binding to hMT1 and hMT2; in vivo evaluation of IOP reduction in rat models	Computational molecular modeling of melatonin and agomelatine binding; in vivo topical administration in hypertensive rats	Compared melatonin vs. agomelatine binding affinity and IOP-lowering effects	Agomelatine had higher stability and affinity for hMT2 compared to melatonin; in vivo, it showed more prolonged IOP reduction.	Agomelatine is a promising melatonin analog with improved stability and longer-lasting IOP-lowering effects, making it a potential therapeutic option for glaucoma.
