# Supplementary material for: Melatonin in Glaucoma: Integrative Mechanisms of Intraocular Pressure Control and Neuroprotection
Source: Biomedicines. 2025 May 16;13(5):1213. doi: 10.3390/biomedicines13051213 (PMC12108883; doi:10.3390/biomedicines13051213)
Supplement: Supplementary file 1 [file biomedicines-13-01213-s001.zip › Supplementary_Table_S2_Neuron_Longtable_Landscape_LeftAligned.pdf]

Supplementary Table S2: Evidence Supporting the Neuroprotective Effects of Melatonin in Glaucoma

Table S2: Evidence Supporting the Neuroprotective Effects of Melatonin in Glaucoma

Author	Study Sample	Methods and intervention	Administration method	Comparison	Variant	Pathway	Outcome	Conclusion
Belforte, Nicolás A et al. J Pineal Res. 2010;48(4):353-364	Male Wistar rats (200 ± 40 g)	Experimental glaucoma induced by weekly intracameral hyaluronic acid (HA) injections; melatonin administration	Subcutaneous melatonin pellet (20 mg with 3% w/v vegetable oil), replaced every 15 days	Vehicle, HA, HA+ Melatonin (administered before or after ocular hypertension induction)	Retinal glutamate clearance, GABA concentrations, nitric oxide (NO) synthesis, oxidative stress markers (SOD, GSH, TBARS), RGC survival, electroretinography (ERG), retinal histology	Melatonin restores glutamate-GABA balance, inhibits NO overproduction, and reduces oxidative stress, preventing glaucomatous neurodegeneration	Melatonin preserved retinal function, protected RGCs, prevented oxidative damage, and reversed functional and histological alterations caused by ocular hypertension	Melatonin exhibits neuroprotective effects in glaucoma by counteracting excitotoxicity, oxidative stress, and inflammation, making it a promising therapeutic candidate for managing glaucomatous neurodegeneration
Zhao, Wen-Jie et al. J Physiol. 2010;588(Pt 14):2605-2619	Sprague-Dawley rats (100–150 g)	Electrophysiological recordings in dissociated RGCs and retinal slices; melatonin administration	Extracellular application of melatonin (10–50 nM)	Control, Melatonin, Melatonin+4-P-PDOT (MT2 receptor antagonist), Melatonin+D609 (PC-PLC inhibitor), Melatonin+Bis IV (PKC inhibitor)	Glycine receptor-mediated currents, intracellular Ca^{2+} levels, protein kinase activation (PKC, PKA, PKG), MT2 receptor expression	Melatonin potentiates glycine currents in RGCs through a phosphatidylcholine (PC)-specific PLC/PKC signaling pathway, independent of cAMP-PKA or cGMP-PKG pathways	Melatonin enhanced glycine receptor-mediated inhibitory currents, improving contrast detection in low-light conditions	Melatonin modulates retinal inhibitory signaling via MT2 receptor activation, enhancing night vision by regulating glycinergic neurotransmission in RGCs
Marangoz, Deniz et al. Int Ophthalmol. 2018;38(6):2553-2562	Wistar albino rats (150–250 g)	Glaucoma model induced by episcleral vein cauterization; brimonidine tartrate (BRT) and melatonin (MEL) administration	Intraperitoneal injection (BRT: 1 mg/kg/day, MEL: 4 mg/kg/day)	Control, Glaucoma, BRT, MEL, Glaucoma+BRT, Glaucoma+MEL	Intraocular pressure (IOP), RGC survival (3% Fluorogold labeling), apoptosis (TUNEL assay)	BRT reduces IOP and protects RGCs; MEL reduces IOP only under glaucomatous conditions but does not prevent RGC apoptosis	BRT significantly reduced IOP and preserved RGCs, while MEL had no significant neuroprotective effect despite lowering IOP	BRT provides neuroprotection against glaucomatous injury, whereas MEL fails to prevent RGC apoptosis, suggesting BRT is superior as a neuroprotective agent in glaucoma
Dal Monte, Massimo et al. Int J Mol Sci. 2020;21(23):9267	Sprague-Dawley rats (8 weeks old)	Methylcellulose-induced hypertensive glaucoma model; topical melatonin/agomelatine eye drops	Topical eye drops	Control, MCE-induced glaucoma, MCE+Timolol, MCE+Brimonidine, MCE+Melatonin/ Agomelatine	Intraocular pressure (IOP), retinal ganglion cell (RGC) survival, electroretinography (ERG), inflammatory markers (GFAP, Iba1, cytokines), apoptotic markers (Bax/Bcl-2, caspase-3)	Melatonin/agomelatine reduces IOP, inhibits gliosis-related inflammation, and prevents RGC apoptosis via Bax-caspase-3 pathway	Melatonin/agomelatine significantly lowered IOP, preserved RGC survival, reduced inflammation, and improved retinal function	Topical melatoninergic compounds show strong hypotensive and neuroprotective effects, offering a potential treatment for hypertensive glaucoma

Continued on next page

Table S2 Continued

Author	Study Sample	Methods and intervention	Administration method	Comparison	Variant	Pathway	Outcome	Conclusion
González Fleitas, María F et al. Mol Neurobiol. 2021;58(8):3653-3664	Male Wistar rats (2 months old)	Experimental glaucoma induced by weekly intracameral chondroitin sulfate (CS) injections; melatonin administration	Subcutaneous melatonin pellets (20 mg with 3% w/v vegetable oil), replaced every 14 days	Vehicle, CS, CS+Melatonin	Melanopsin-expressing RGC number, anterograde retinal transport to SCN and OPN, pupillary light reflex, light-induced c-Fos expression, locomotor activity rhythm, mitochondrial markers (TOM20, SOD2)	Melatonin prevents mitochondrial dysfunction in melanopsin-expressing RGCs, preserving non-image-forming visual system integrity	Melatonin preserved melanopsin-expressing RGCs, maintained retinal transport to SCN and OPN, improved light-evoked pupil constriction, restored locomotor rhythm, and prevented mitochondrial alterations	Melatonin protects the non-image-forming visual system against glaucoma-induced damage, likely through a mitochondrial protective mechanism, highlighting its potential for circadian and neuroprotective interventions in glaucoma
Zhang, Yu et al. CNS Neurol Disord Drug Targets. 2021;20(3):285-297	Rat model of acute high intraocular pressure (HIOP)	Induction of HIOP by increasing intraocular pressure followed by reperfusion; melatonin administration	Not specified	Control, HIOP, HIOP+Melatonin	Pyroptosis markers (GSDMD, GASMDp32, Caspase-1, Caspase-1 p20), inflammatory cytokines (IL-18, IL-1β), cell death (Ethidium Homodimer III staining, LDH release)	Melatonin prevents pyroptosis through the NF-$\kappa\beta$ /NLRP3 axis	Melatonin reduced pyroptosis in retinal neurons, especially in the ganglion cell layer, and decreased inflammation after acute HIOP injury	Melatonin may serve as a potential neuroprotective agent against retinal pyroptosis following acute HIOP injury
Wang, Chao et al. Front Endocrinol (Lausanne). 2022;13:986131	R28 retinal precursor cells and C57BL/6 mice (8 weeks old)	Glutamate-induced excitotoxicity in R28 cells; NMDA-induced retinal damage in mice; melatonin administration	Intravitreal injection	Control, Glu-treated, Glu+Melatonin, NMDA-treated, NMDA+Melatonin	R28 cell viability, reactive oxygen species (ROS), glutathione (GSH) levels, retinal ganglion cell (RGC) survival, visual function (FVEP), RNA sequencing	Melatonin mitigates glutamate excitotoxicity and NMDA-induced damage via reducing oxidative stress, preserving retinal transcriptome integrity, and modulating PI3K-AKT and JAK-STAT pathways	Melatonin reduced RGC death, improved cell viability, lowered oxidative stress, preserved retinal structure, and maintained visual function	Melatonin offers neuroprotection against glutamate excitotoxicity and NMDA-induced retinal injury, supporting its potential role in glaucoma treatment
Ye, Dan et al. J Pineal Res. 2022;73(4):e12828	C57BL/6J mice (6–8 weeks old)	Acute ocular hypertension (AOH) model induced by elevating IOP; melatonin administration	Intraperitoneal injection (20 mg/kg/day)	Sham, Sham+Melatonin, AOH, AOH+Melatonin	Retinal thickness, RGC survival, ERG amplitudes, apoptotic markers (caspase-3, Bax, Bcl-2), necroptosis markers (RIP1, RIP3, MLKL), pyroptosis markers (NLRP3, ASC, caspase-1, GSDMD)	Melatonin inhibits PANoptosis (apoptosis, necroptosis, pyroptosis) by downregulating caspase-3, RIP1/RIP3, and NLRP3 pathways	Melatonin preserved retinal structure and function, reduced inflammatory response, and promoted RGC survival	Melatonin exerts neuroprotection in AOH injury by inhibiting PANoptosis, highlighting its potential as a therapeutic agent for acute glaucoma

Continued on next page

Table S2 Continued

Author	Study Sample	Methods and intervention	Administration method	Comparison	Variant	Pathway	Outcome	Conclusion
Zhang, Fan et al. Antioxidants (Basel). 2023;12(6):1173	C57BL/6J male mice (6–8 weeks old)	Induction of retinal ischemia-reperfusion (RIR) injury via acute ocular hypertension model; melatonin administration	Intravitreal injection	Sham, RIR+Vehicle, RIR+Melatonin (low/high doses), RIR+Ferrostatin-1	Retinal ganglion cell (RGC) survival, lipid peroxidation, ferroptosis markers (Slc7a11, Fth1, Alox12, p53), inflammatory markers (IL-1β, IL-18), mitochondrial morphology	Melatonin inhibits p53-mediated ferroptosis via Slc7a11/Alox12 axis, reducing lipid peroxidation and oxidative stress	Melatonin significantly reduced RGC death, lipid peroxidation, iron accumulation, and inflammation while preserving mitochondrial structure	Melatonin confers neuroprotection in RIR injury by inhibiting p53-mediated ferroptosis, reducing oxidative stress and inflammation, suggesting a potential therapy for retinal neuroprotection
Zou, Jingling et al. Int Immunopharmacol. 2023;118:109976	C57BL/6J mice (8 weeks old)	NMDA-induced retinal ganglion cell (RGC) injury model; melatonin administration	Intraperitoneal injection (20 mg/kg/day)	Control, NMDA, NMDA+Melatonin, NMDA+PLX3397 (microglia inhibitor), NMDA+Infliximab (TNFα inhibitor), NMDA+SB202190 (p38 MAPK inhibitor)	RGC survival, microglial activation markers (Iba1, CD68), inflammatory cytokines (TNFα, IL-1β, NOS2, IL-17), apoptotic markers (TUNEL), p38 MAPK activation	Melatonin suppresses microglia-derived TNFα production, inhibiting the RGC p38 MAPK pathway and reducing neuroinflammation	Melatonin preserved RGC survival, reduced microglial activation and TNFα levels, inhibited p38 MAPK activation, and improved retinal function	Melatonin exerts neuroprotection in NMDA-induced RGC injury by regulating the microglia-TNFα-RGC p38 MAPK pathway, highlighting its potential for retinal neurodegenerative diseases like glaucoma
Gao, Shan et al. Mol Cell Biochem. 2024;479(12):3393-3404	RGC-5 retinal ganglion cells (immortalized cell line)	H2O2-induced oxidative stress in RGC-5 cells; melatonin pretreatment	Melatonin (10 μM) pre-treatment for 3 hours before H2O2 exposure	Control, H2O2-treated, H2O2+Melatonin, Trx1 knockdown, Trx1 knockdown+Melatonin	Cell viability, apoptosis markers (cleaved-caspase 3, cleaved-caspase 9, Bax, Bcl-2), oxidative stress markers (ROS, LDH, MDA), Trx1 pathway activation	Melatonin reduces oxidative stress-induced apoptosis by activating the Trx1-mediated JNK/ERK/p38 MAPK pathway	Melatonin improved cell viability, reduced ROS accumulation, restored Trx1 expression, and inhibited apoptosis in oxidative stress-damaged RGC-5 cells	Melatonin protects RGCs from oxidative stress by modulating the Trx1 signaling pathway, suggesting its potential therapeutic role in retinal neurodegenerative diseases like glaucoma
Shi, Yuxun et al. Biochim Biophys Acta Mol Basis Dis. 2024;1870(4):167053	C57BL/6J mice (6–8 weeks old)	Optic nerve crush (ONC) model; melatonin administration	Intraperitoneal injection (20 mg/kg/day)	Sham, Sham+Melatonin, ONC, ONC+Melatonin, ONC+Melatonin+EX-527 (SIRT1 inhibitor)	RGC survival, ganglion cell complex (GCC) thickness, p-ERG amplitudes, senescence markers (p21, SA-β-gal), apoptosis markers (TUNEL, caspase-3)	Melatonin attenuates RGC senescence and apoptosis via a SIRT1-dependent mechanism by modulating p53 acetylation and reducing oxidative stress	Melatonin preserved RGC survival, maintained GCC thickness, reduced senescence and apoptosis, and improved retinal function	Melatonin exerts neuroprotection against ONC-induced RGC damage by activating SIRT1, highlighting its potential as a treatment for neurodegenerative retinal diseases like glaucoma

Continued on next page

Table S2 Continued

**Author**	**Study Sample**	**Methods and intervention**	**Administration method**	**Comparison**	**Variant**	**Pathway**	**Outcome**	**Conclusion**
Hu, Chenyang et al. J Pineal Res. 2024;76(1)	C57BL/6J mice (wild-type) and EAAC1 knockout (EAAC1−/−) mice	EAAC1 deletion-induced retinal ganglion cell degeneration model; melatonin administration	Daily oral administration (5 µg/mL in drinking water) from 5 weeks old to 6 months	WT+Vehicle, WT+Melatonin, EAAC1−/−+Vehicle, EAAC1−/−+Melatonin	Retinal morphology (SD-OCT, HE staining), RGC survival, oxidative stress markers (ROS, GSH), apoptosis markers (TUNEL, Bax, Bcl-2), senescence markers (p16, γ-H2AX), inflammation markers (CD45, Iba1, CD68), retinal function (ERG)	Melatonin inhibits oxidative stress-induced apoptosis and senescence by modulating NRF2/p53/Sirt1 signaling pathways	Melatonin preserved retinal structure, reduced oxidative stress, inhibited apoptosis and senescence, and improved retinal function	Melatonin provides neuroprotection against EAAC1 deletion-induced RGC degeneration by reducing oxidative stress and modulating NRF2/p53/Sirt1 pathways, highlighting its potential for normal tension glaucoma treatment
